# Do Pentraxins Bind to Fungi in Invasive Human Gastrointestinal Candidiasis?

**DOI:** 10.3390/jof4030111

**Published:** 2018-09-17

**Authors:** Umamaheshwari Golconda, Richard E. Sobonya, Stephen A. Klotz

**Affiliations:** 1 Department of Pathology, University of Arizona College of Medicine, Tucson, AZ 85724, USA; umagolgonda@email.arizona.edu (U.G.); rsobonya@email.arizona.edu (R.E.S.); 2 Division of Infections Diseases, Department of Medicine, University of Arizona College of Medicine, Tucson, AZ 85724, USA

**Keywords:** *Candida*, serum amyloid P component, C-reactive protein, pentraxin 3, pentraxins, amyloid, fungi, immunohistochemistry

## Abstract

Tissue from 13 autopsy cases with invasive gastrointestinal candidiasis was studied for the binding of the pentraxins, C-reactive protein (CRP), pentraxin 3 (PTX3), and serum amyloid P component (SAP) to fungal surfaces. Invasive candidal infection was demonstrated using a hematoxylin and eosin stain and a Gomori methenamine silver stain (GMS). Immunohistochemistry was performed with CRP and PTX3 monoclonal antibodies and did not demonstrate CRP or PTX3 bound to fungi (0 of 13 cases), although CRP was extensively deposited on human tissue. A polyclonal antibody to SAP showed that SAP was bound to fungi in 12 of 13 cases. Although all three pentraxins have been reported to bind to fungi or bacteria, only SAP was bound to filamentous and yeast forms of *Candida* in human tissue, as detected by immunohistochemistry. SAP was abundantly present on fungi and may have affected the host innate immune response to the invading fungi.

## 1. Introduction

Pentraxins are a family of proteins that play an important and integral role in innate immunity. The best characterized pentraxins are C-reactive protein (CRP), pentraxin 3 (PTX3), and serum amyloid P component (SAP). CRP and SAP (known as short pentraxins based on the primary structure of their subunit) are made in the liver and circulate in the serum, whereas PTX3 is secreted at the local level by numerous cells in response to inflammation. CRP and SAP share an annular shape possessing identical homopentamers, whereas PTX3 (known as a long pentraxin) has a longer N-terminal protein subunit [1]. These proteins have been likened to ancestral antibodies [2]. They are pattern recognizing proteins believed to be important in identifying harmful microbes and helpful in preparing molecules for removal or neutralizing their effects in the host [2].

We have found that one pentraxin is detectable on fungi in invasive human diseases. Fungal adhesins, such as Als5p, form amyloid-like nanodomains on the surface of fungal cells, both in vitro [3] and in vivo in invasive fungal infections [4,5,6]. Fungal cells with these nanodomains bind SAP [4]. Since the chance discovery of SAP binding to fungal structures in human disease [4] we have not only been interested in SAP-fungal interactions, but whether pattern recognizing proteins such as other pentraxins interact with *Candida* as well. Accordingly, we attempted to detect CRP, PTX3, and SAP on fungi invading human gastrointestinal tissue.

## 2. Methods

### 2.1. Staining Human Tissue

We examined stored autopsy specimens from 13 patients with histological evidence of invasive candidiasis of the gastrointestinal tract. These cases, with the exception of one new case, were included in our 2012 report [4]. The antibody to SAP, however, is different from what we used before. Tissue blocks were cut thinly, deparaffinzed, and stained with hemotoxylin-eosin (H&E). Multiple H&E-stained slides of infected material were carefully examined for the presence of polymorphonuclear leukocytes (PMNs) and other inflammatory response cells (lymphocytes) in tissue adjacent to yeasts and filamentous forms. They were designated as 0: (no inflammatory cells seen); 1+: minimal (none to almost no cells seen), 2+: moderate (moderate number of cells seen); or 3+: brisk (cells too numerous to count). Cell counts were made by two pathologists. Slide sections found to be positive for *Candida* species (presence of yeasts, pseudohyphae, and/or hyphae by microscopy) were then stained with Gomori methenamine silver (GMS).

### 2.2. Immunohistochemistry

Tissue from the 13 autopsy cases underwent a citrate antigen retrieval for 20 min at 97 °C. Mouse monoclonal antibody with CRP or PTX 3 or a rabbit polyclonal antibody with SAP (all Sigma-Aldrich, St. Louis, MO, USA) were diluted 1/300 in Tris-buffered saline, and added along with surfactant, protein stabilizer, and 0.35 ProClin 950 (Sigma-Aldrich) for 30 min. The secondary antibodies were anti-mouse or anti-rabbit IgG (Sigma-Aldrich) and diluted in Tris-buffered saline containing 10% animal serum and 0.09% ProClin 950 from Leica’s Refine Detection Kit (Leica Biosystems Newcastle Ltd., Newcastle Upon Tyne, UK). The secondary antibody was tagged with horseradish peroxidase, either brown or red. The brown antibody reactions were difficult to interpret in some cases, particularly the CRP antibody, because of lipofuchsin present in the tissue (it appears brown on light microscopy). Therefore, with CRP antibodies we measured the binding of antibodies to fungi with the red reaction only. Human tissue that tested positive for CRP, PTX3, and SAP were kidney, prostate and kidney, respectively.

### 2.3. Clinical Information

The clinical data used to compile Table 1 was extracted from the Autopsy Summaries of the 13 cases. Blood cultures represent heart blood samples taken at autopsy, a reliable indicator of disseminated candidiasis [7]. *Candida* isolated from tissue was not always identified to species level and was often reported as “yeasts” only.

## 3. Results

**Figure 1:**
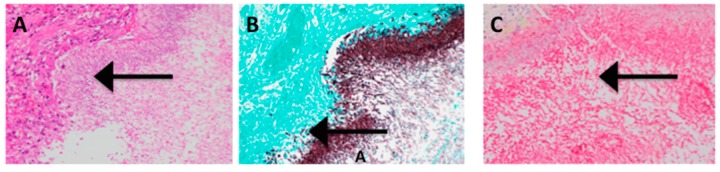
(**A**) Hematoxylin and Eosin stain of esophagus, with a large ulceration of the lumen and a loss of epithelium (200×). Massive proliferation of *Candida* yeasts and filamentous forms (indicated with arrow: fungi appear violet-pink). There was only minimal cellular response to the infection. The patient died of a large bleeding ulcer in the stomach and had no history of immunosuppression. (**B**) Gomori methenamine silver (GMS) stain in the identical area as A (the host tissue stains blue-green) demonstrating fungi (blackish red) invading tissue (indicated with arrow) (200×). (**C**) Same area as B, stained with antibody to SAP. The yeasts and filamentous forms of *Candida* were coated with antibody. In the upper left corner was human tissue not stained with antibody (~300×).

**Figure 2:**
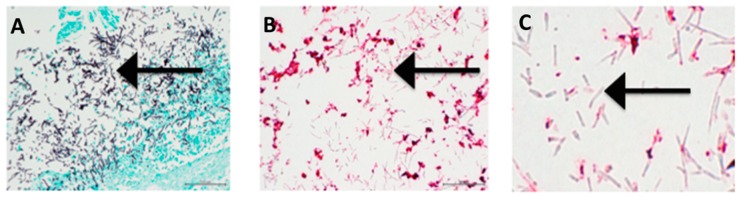
(**A**) GMS stain showing fungi (black, indicated with arrow) invading gastric tissue (green). The patient died of acute myeloid leukemia. Post mortem blood cultures yielded *Candida*, *Enterococcus* and *Xanthomonas* species (200×). (**B**) Yeast cells and filamentous forms of *Candida* (arrow) in same area as A, but stained with antibody to CRP. There was CRP decorating human cellular debris, but the fungi did not stain (200×). (**C**) Enlargement of B, the fungi were clearly translucent and not staining (indicated with arrow), whereas cellular debris was stained with antibody to CRP (400×).

**Figure 3:**
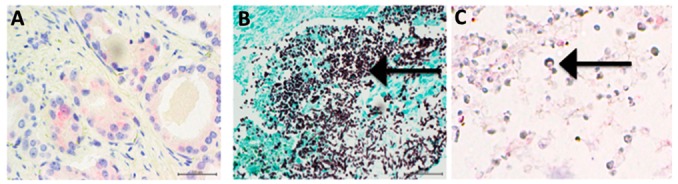
(**A**) Prostate tissue stained for PTX3. This tissue was rich in PTX3, tubular cells, and secretions were stained a rose color (400×). (**B**) Area adjacent to Figure 2A, stained with GMS, showing large amount of fungi (black) (200×). (**C**) Same area as B, stained for PTX3. There are numerous yeast cells seen (arrow) and ghosts of filamentous forms of *Candida* that are translucent and not stained by antibody to PTX3 (400×).

All 13 cases demonstrated the presence of *Candida* invading tissue on H&E stain (Figure 1A). Abundant fungi, both yeasts and filamentous forms were demonstrated using a GMS stain (Figure 1B). Areas with fungi on GMS and H&E stained tissue were carefully observed in antibody stained tissue slides. Figure 1C shows that SAP is abundantly present on fungal cells whereas, Figure 2B,C and Figure 3C show that neither CRP nor PTX3, respectively, were found on fungal cells.

Table 1 tabulates the pathologic findings and microbiology data of the 13 cases. No premortem serum CRP levels were available in these cases. In circumstances where one would anticipate very high CRP levels, such as cases of candidemia, no CRP was demonstrated bound to fungi, nor in any other case. Many of the patients had severe erosion and invasion of the esophagus by *Candida.* Two cases had what was described as non-invasive *Candida* infections on the esophagus with an abundance of yeasts and hyphae, but no invasion of mucosa. In our prior work with these cases, we determined that the host inflammatory response was negligible even in the presence of elevated peripheral white blood cell counts [4]. In our present study, however, it was apparent that polymorphonuclear leukocytes were absent or scant in most cases; there were two cases with 2+ PMNs (moderate) and one case with 3+ PMNs (brisk) (Table 1). Similarly, the number of lymphocytes or mononuclear cells were judged to be insufficient. Only 4 of 13 of the cases had a presumed reduction in cellular immunity due to disease or treatment regimens.

The flow cytometry of *C. albicans,* incubated with CRP at 50 μg/mL, showed a negligible binding of CRP, only 7% of fungi bound CRP. By contrast, SAP at 30 μg/mL bound to 80–100% of fungi (manuscript in preparation). The inability of CRP to bind in large amounts to *Candida* in the flow cytometry correlated with our immunohistochemistry findings reported here with CRP.

## 4. Discussion

The binding of molecules of innate immunity to fungi may affect the innate immune response of macrophages and polymorphonuclear leucocytes in interactions with these fungi. For example, SAP binds to macrophages and down-regulates host response by increasing IL-10 secretion and can even ameliorate the fibrotic response [8]. SAP binds avidly to amyloid fibrils, DNA, and many microbes [1]. CRP, on the other hand, is an inflammatory marker and an acute phase reactant. Its discovery involved the finding that CRP precipitated C-polysaccharide from serum. C-polysaccharide is found in the capsule of *Streptococcus pneumoniae* [9] and the ligand of CRP within C-polysaccharide is phosphocholine [10]. The third pentraxin, PTX3, appears to play a critical role in infections with *Aspergillus*. It binds to conidia of *Aspergillus* and in so doing ameliorates pulmonary infection in mice caused by this fungus [11].

However, PTX3 does not bind to *C. albicans* yeast cells when studied with flow cytometry [11] or with immunohistochemistry (our data). The same pertains for CRP—i.e., it neither binds to *Candida albicans* yeasts when measured with flow cytometry nor with immunohistochemistry. Conversely, serum amyloid P component coats *Candida* yeasts and the filamentous forms invading human tissue, as demonstrated by immunohistochemistry or flow cytometry (manuscript in preparation). In previous work, we established that SAP binds to the *Candida* cell surface functional amyloid [4]. We demonstrated the same phenomenon in invasive human cases of *Aspergillus*, *Coccidioides*, and Mucorales [6]. SAP binds to many microbes including parasites, bacteria, viruses, and fungi and this binding may be explained by the presence of functional amyloid on the surface of the microbes [12]. These SAP-coated microbes may dampen the immune response to infection [13].

SAP has several ligands, including amyloid fibrils [14] and phosphoethanolamine [15] which may be present in the cell membranes of *Candida albicans* [16]. SAP also binds to phosphocholine but less avidly than to phosphoethanolamine [17]. Our previous results imply that amyloid formation per se is the trigger for SAP binding, as the amyloid-like structures are high avidity ligands found on *Candida* [4]. In any case, further work is needed to show the specificity of this binding as well as its potential interaction with the immune response.

The failure to detect CRP on fungi was unexpected. As mentioned previously one ligand of CRP is phosphocholine [10] and it can be exposed in fungi as it is present in the cell walls of *Saccharomyces*, *Candida* and other fungi as part of the GPI anchor of adhesin molecules [18]. Moreover, CRP is measurable in the serum and present in many different pathological conditions where sometimes it is pro-inflammatory and at other times, anti-inflammatory [19]. Furthermore, CRP appeared to opsonize *C. albicans* for phagocytosis by polymorphonuclear leukocytes [20].

PTX3 can be measured in plasma in nanogram amounts in patients with sepsis and shock and if measured serially, may have utility in risk assessment in sepsis and shock [21]. However, we were unable to detect it bound to *Candida*, a feature that has been reported before [11]. The absence of PTX3 around the fungi may be due to the poor cellular response to invading fungi in the tissues [4].

Since these protein deposits on fungi could occur non-specifically following death of the patient we sought to determine if such might be the case. All three of these pentraxins are reported in autopsy material [22,23,24] and the binding patterns were similar to biopsy specimens involving living tissue. CRP appears to demonstrate several specific patterns in autopsy material, not just one pattern [22]. It is unlikely these findings reported here represent non-specific coating of tissue and pathogens in the case of SAP.

## 5. Conclusions

Of the three major pentraxins, only the serum amyloid P component bound avidly and abundantly to the *Candida* cell surfaces invading human tissue (12 of 13 autopsy cases). No binding was detected for CRP or PTX3 to fungal surfaces. The presence of SAP on the fungi in the tissue may have an effect upon the host response to the infection [13].

## Figures and Tables

**Table 1:** Clinical and microscopic findings of 13 gastrointestinal candidiasis autopsy cases.

Candidal Lesion(s) *	Reduced Cellular Immunity	Cause of Death; Associated Problem(s)	Microbiology Cultures **	Serum Amyloid P (SAP) on Fungi	Pentraxin 3 (PTX 3) on Fungi	C-Reactive Protein (CRP) on Fungi	PMN in Lesion ***	Lymph cells in Lesion ***
Esophagitis; myocarditis encephalitis	No	Prematurity necrotizing enterocolitis; disseminated candididasis	*C. albicans*, *E. coli* in blood	+	−	−	1+	1+
Esophagitis	No	Ischemic heart disease	Group D *Streptococcus* in blood	+	−	−	0	0
Esophagitis, gastritis	No	Sepsis; non-Hodgkins	*C. albicans*, *Enterococcus* in blood	+	−	−	1+	1+
Esophagitis, gastritis	No	Myocardial infarction	Yeast in lung (only yeasts were found in tissue)	-	−	−	2+	2+
Esophagitis	No	Pulmonary embolism		+	−	−	1+	1+
Gastric ulcer	Yes	Sepsis; exsanguination; treated acute myelomonocytic leukemia	*Candida* sp., *Enterococcus*, *Xanthomonas maltophilia* in blood	+	−	−	0	1+
Esophagitis	Yes	Disseminated aspergillosis; pneumonitis; pancytopenia; acute myeloid leukemia		+	−	−	2+	1+
Esophagitis	Yes	Hemorrhagic enterocolitis; acute lymphocytic leukemia; graft versus host disease	Disseminated aspergillosis	+	−	−	0	1+
Colitis	No	Sepsis; pseudomembranous colitis	*C. krusei*, Group D *Streptococcus* in blood; *Aspergillus* in tissue	+	−	−	0	1+
Gastric ulcer	No	Gastrointestinal hemorrhage; gastric ulcer		+	−	−	3+	1+
Esophagitis	No	Ischemic heart disease		+	−	−	0	0
Duodenal ulcer	No	Exsanguination from peptic ulcer	*C. albicans* in blood; yeasts in lung	+	−	−	1+	1+
Esophagitis	Yes	Sepsis; neutropenia; post-operative cancer resection of colon	*Escherichia coli*; viridans *Streptococcus* in blood; yeasts in lung	+	−	−	0	1+

*: organ or site where *Candida* was found microscopically; **: some cultures were not speciated and were denoted as yeasts when positive; +/−: positive or negative immunohistochemistry results; ***: PMN: polymorphonuclear leukocyte; numerical scoring of tissue: 0: no host cells seen within lesion or adjacent to fungi; 1+: minimal cells seen; 2+: moderate numbers of cells seen; 3+: too numerous to count cellular infiltrate.
